# Comparison of multistranded wire and fiber-reinforced composite retainers effects on periodontium: A randomized clinical trial

**DOI:** 10.1590/2177-6709.28.1.e2319380.oar

**Published:** 2023-04-03

**Authors:** Nasreen Iqbal NAGANI, Imtiaz AHMED

**Affiliations:** 1 Dow University of Health Sciences Karachi, Dr. Ishrat-Ul-Ebad Khan Institute of Oral Health Sciences (DIKIOHS), Department of Orthodontics (Karachi/Pakistan).

**Keywords:** Fixed retainers, Orthodontic wires, Clinical trial

## Abstract

**Introduction:**

Fixed orthodontic retainers are very important for treatment stability; however, adverse effects on the health of periodontium can be caused as a result of deposition of plaque and calculus.

**Objectives:**

To compare and determine the effects of two mandibular fixed lingual retainers on the periodontal status, and to test the null hypothesis that there would be no significant difference on the periodontium health between the patients using fiber-reinforced composite (FRC) or multistranded wire (MSW) fixed retainers.

**Methods:**

A total of 60 subjects were recruited, out of which 6 were excluded and 2 dropped out during the study. Hence, 52 subjects with mean age of 21.5 ± 3.6 years were included in the study. The sample was composed by 8 males (15.4%) and 44 females (84.6%). The participants were randomly divided into two groups: Group 1 received fiber-reinforced composite retainer, while Group 2 received multistranded wire retainer. After insertion, plaque index, calculus index, gingival index and bleeding on probing were compared, after three months (T1), six months (T2), nine months (T3) and twelve months (T4), using Mann-Whitney test with *p*-value ≤ 0.05 as significant.

**Results:**

It could be seen that the health of periodontium deteriorated with the passage of time from T1 to T4 in both group of retainers. However, no statistically significant differences were found between the two groups (*p*> 0.05).

**Conclusion:**

The results of the study indicate that there was no significant difference on the health of periodontium between the patients with FRC and MSW fixed retainers, hence, the null hypothesis was accepted.

## RESUMO

**Introdução:**

As contenções ortodônticas fixas são muito importantes para a estabilidade do tratamento; no entanto, elas podem causar efeitos adversos na saúde do periodonto, como resultado da deposição de placa e cálculo.

**Objetivos:**

Comparar e determinar os efeitos na saúde periodontal de duas contenções inferiores coladas por lingual, e testar a hipótese nula de que não haveria diferença significativa na saúde periodontal entre os pacientes usando contenções fixas de compósito reforçado com fibra (FRC) ou de fio multitrançado (MSW).

**Métodos:**

No total, 60 indivíduos foram recrutados, dos quais 6 foram excluídos e 2 desistiram durante o estudo. Assim, foram incluídos no estudo 52 indivíduos com média de idade de 21,5 ± 3,6 anos. A amostra foi composta por 8 homens (15,4%) e 44 mulheres (84,6%). Os participantes foram divididos aleatoriamente em dois grupos: O Grupo 1 recebeu contenção de compósito reforçado com fibra, enquanto o Grupo 2 recebeu contenção de fio multitrançado. Após três meses (T1), seis meses (T2), nove meses (T3) e doze meses (T4) da colagem, foram comparados os índices de placa e de cálculo, índice gengival e sangramento à sondagem, usando o teste de Mann-Whitney com *p* ≤ 0,05 como significativo.

**Resultados:**

Pôde-se observar que a saúde periodontal piorou com o passar do tempo, de T1 a T4, em ambos os grupos de contenções. No entanto, não foram encontradas diferenças estatisticamente significativas entre os dois grupos (*p*> 0,05).

**Conclusão:**

Os resultados do estudo indicam que não houve diferença significativa na saúde periodontal entre os pacientes com contenções fixas FRC e MSW; portanto, a hipótese nula foi aceita.

## INTRODUCTION

After the completion of the fixed orthodontic treatment, outcome stability is assumed as a great challlenge.^1^ It is essential to maintain the teeth on the newly acquired position, thus allowing periodontal and gingival fibers reorganization to prevent relapse.^2^ With this regard, orthodontic retainers are bonded at the end of the treatment, being considered a mandatory procedure to several authors.^3^

Fixed retainers provide retention for prolonged time, and are the most common choice for the mandibular arch.^4^ Multistranded spiral wire retainers (MSW) are flexible in nature and are considered to be the gold standard in providing optimal fixed retention in contemporary orthodontics.^5^ Efficacy and reliability of these retainers bonded on the lingual surfaces of anterior teeth have been reported in several studies.^4^ ^,^ ^5^

Fiber-reinforced composites (FRC) are innovative materials with excellent splinting properties, composed of multiple fibers made up of carbon, polyaramid, polyethylene and glass.^6^ They are indicated for many applications, such as periodontal splinting, fixed orthodontic retainers, restorations, endodontic post and cores, and bridges.^7^ ^,^ ^8^ FRCs are bonded by chemical adhesion due to the presence of oxygen-inhibited layer on its outer surface, which results in direct chemical bonding with the composite and micromechanical retention of composite resin with the tooth surface.^9^ FRCs transmit the forces to the glass fibers, thus strengthening the resistance offered by the bonding agent.^10^ Moreover, the difference of physical properties in the bonding interface of the two materials (composite resin and wire) is also eliminated.^11^ FRCs are well tolerated by the patients^12^ and present good biocompatibility, especially for the nickel allergic patients.^2^ ^,^ ^13^ FRCs do not request great amount of material for installation and are easily contoured along the lingual surfaces of the teeth. FRCs also offer enhanced esthetics and allow easy repair in cases of damage. However, the resin material allows limited physiological tooth movement, as they create a rigid splint, which may lead to bond failure.^14^

Despite of all the gains, fixed retainers are criticized for causing adverse effects on the health of periodontium due to the accumulation of plaque and calculus.^15^ ^,^ ^16^ Multiple studies have been found in literature evaluating the effects of various stainless steel retainers on the health of periodontium,^15^ ^,^ ^17^ ^,^ ^18^ ^,^ ^19^ but clinical follow-up studies on the efficacy of newly introduced fiber-reinforced composite are scarce. Thus, the objectives of this study were to compare and determine the effects of two mandibular fixed lingual retainers on the periodontal status.

Thus, the present study tested the null hypothesis that there would be no significant difference in the health of periodontium between the patients with FRC and MSW fixed retainers.

## MATERIAL AND METHODS

This parallel-group randomized clinical trial, multicenter study with a 1:1 allocation ratio, registered under the protocol ID NCT03881813 (https://clinicaltrials.gov/), according to the CONSORT statement of the updated guidelines for reporting randomized clinical trials, was approved by the Institutional Review Board of the university (Ref:IRB-941/DUHS/Approval/2017/162). The study was conducted at the department of Orthodontics, Dr. Ishrat Ul Ebad Khan Institute of Oral Health Sciences (DIKIOHS) and Dow Dental College (DDC), in Dow University of Health Sciences (DUHS), from November 2017 to March 2019.

**Figure 1:**
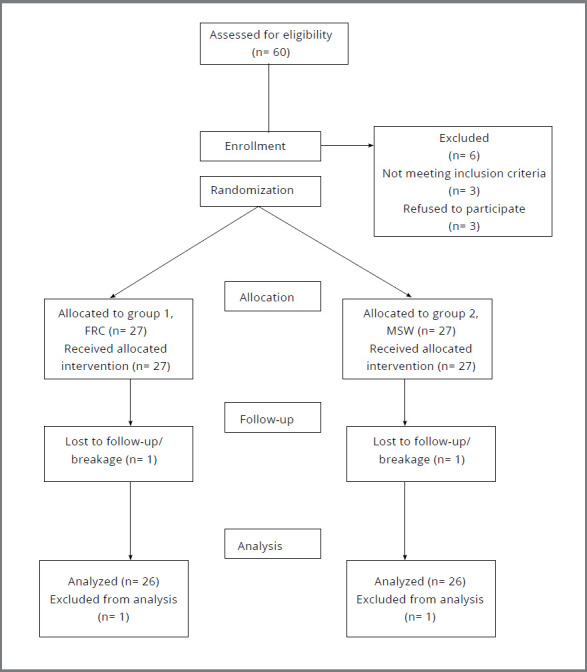
Flow diagram showing distribution of subjects.

The sample size was calculated with PASS (v.11) using two sample proportion with 95% confidence interval and 80% power of test, based on the values for measured clinical indices obtained from a previous article with estimated population size of 60 patients in six months^20^ (Fig 1). Convenience sampling technique was followed. Subjects were randomly divided through computer generated software into two groups. Non extraction cases completed fixed orthodontic treatment (MBT, 0.022-in slot) with healthy periodontium and good oral hygiene were included, while subjects with congenitally missing or extracted mandibular anterior teeth were excluded. 

Written informed consents were obtained from all the subjects. After completion of treatment and debonding of brackets, deep scaling and curettage was performed. Then, two types of fixed mandibular lingual retainers from canine-canine were installed. All retainers were bonded by a single operator, to minimize bias and to standardize the method. Blinding was not possible in this study due to the visible nature of the intervention and assessment. Group 1 subjects received fiber-reinforced composite retainers (INOD, U.P. Fiber Splint, 2mm, Seoul, Korea) while Group 2 (control group) received multistranded stainless steel wire retainers (0.0175-in, All Star Orthodontics, Camas, WA, USA).

**Figure 2:**
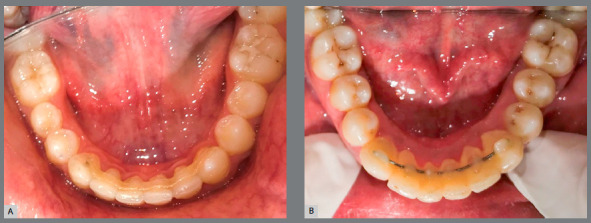
A) Fiber-reinforced composite retainer bonded on the lingual surface of mandibular anterior teeth. B) Multistranded stainless steel wire retainer bonded on the lingual surface of mandibular anterior teeth.

In Group 1 subjects, the mandibular anterior dental region was well isolated. Intercanine distance was measured using dental floss, and the correct length of fiber ribbon was cut with a scalpel blade. The ribbon was pretreated with adhesive primer (Transbond™ XT adhesive primer; 3M Unitek). Lingual surfaces of the six anterior teeth were etched with 37% phosphoric acid gel (Meta Biomed) for 30 seconds, washed sufficiently and air-dried. Then, adhesive primer was applied with applicator brush and light-cured with a light emitting diode (Otholux; 3M) for 15 seconds on each tooth. Subsequently, application of low viscosity composite resin (Transbond™ LV; 3M Unitek, Monrovia, California, USA) was performed. Eventually, fiber ribbon was conformed to the lingual surfaces of the six anterior teeth with plastic instrument, the excess composite was removed, and each tooth was light-cured for 15 seconds. Further composite resin was applied with applicator for finishing. Finally, each tooth was light-cured for 10 seconds. Oral hygiene instructions were delivered (Fig 2A). For the Group 2 subjects (multistranded SS retainer), the same isolation and bonding protocols were followed (Fig 2B).

The subjects were called back at every three months interval, for a period of 12 months: three months (T1), six months (T2), nine months (T3) and twelve months (T4). At every visit, the following indices were evaluated on each subject, by the principal investigator and then was reevaluated by two colleagues to avoid errors, and the mean value was recorded.

» Plaque index (PI), was assessed according to the Plaqsearch (Oraldent Ltd) on lingual surfaces of mandibular anterior teeth, as follows:^21^

0 - Absence or no plaque accumulation.
1 - Plaque detected on probing the gingival margin.
2 - Visible plaque.
3 - Copious plaque.

» Calculus index (CI) was evaluated using the scale, as follows:^22^

0 - No calculus detected.
1 - Calculus seen and covering no more than one third of the tooth surface.
2 - Calculus seen and covering no more than two third of the tooth surface and/or the presence of separate specks of calculus subgingivally.
3 - Calculus seen and covering more than two third of the tooth surface and/or presence of a uninterrupted band of calculus subgingivally.

» Gingival index (GI) was assessed based on the following scale:^21^

0 - No inflammation.
1 - Mild inflammation with minor alteration in color, slight alteration in surface, no bleeding detected on probing.
2 - Moderate inflammation, reasonable glazing, redness, edema and hypertrophy, bleeding detected on probing.
3 - Severe inflammation, noticeable redness, hypertrophy, affinity for unprompted bleeding, ulceration.

» Bleeding on probing (BOP) was assessed 10-15 seconds after the placement of a Florida probe into the gingival crevice, and was evaluated using the following score:^23^

0 - Absence of bleeding.
1 - Presence of bleeding on probing.

These variables were evaluated along the lingual surfaces of the mandibular anterior teeth. Average value was calculated for all mandibular six anterior teeth, and the mean was recorded for each individual on every visit.

## STATISTICAL ANALYSIS

Shapiro-Wilk test was used to check the normality of the data. The data was found to be non-normal. Baseline characteristics between the two groups of retainers were investigated using descriptive statistics. P-values were calculated using Independent t-test and Chi-square test. Due to the ordinal data, the four indices were compared and evaluated between two groups of retainers using Mann Whitney test. SPSS v. 21 was used for all data analysis, and the level of significance for all analysis was set to 0.05.

## RESULTS

A total of 60 subjects were recruited, out of which 6 were excluded (3 were not meeting the inclusion criteria, while 3 refused to participate). Out of 54 subjects, two dropped out during the study, either because they missed appointment or retainer fracture. Subjects with dislodged or broken retainers had to be excluded to minimize bias, as they were referred immediately for the appliance repair to maintain the stability of the dentition. Hence data was analyzed on 52 subjects (26 in each group) (Fig 1).

The measurement error and reliability between the three observers was checked using intraclass correlation, and good agreement was found between the measurements (ICC > 0.70).

The mean age of subjects was 21.5 ± 3.6 years, ranging from 14 to 30 years. Out of 52 subjects, 8 (15.4%) were males, while 44 (84.6%) were females. Out of 52 cases, 38 (73.1%) were Class I malocclusion treated while 14 (26.9%) were Class II treated cases. Table 1 shows the comparison of baseline characteristics between the two groups of retainers. There were no significant differences in the baseline characteristics, i.e. age, sex and type of malocclusion, between the two groups.

**Table 1:** Comparison of baseline characteristics between two groups of retainers.

Baseline characteristics	Group 1 (FRC retainer)	Group 2 (MSW retainer)	p-value *
Age			
mean ± SD	20.88 ± 3.45	22.15 ± 3.68	>0.05
Sex			
Male n (%)	4 (50.0)	4 (50.0)	>0.05
Female n (%)	22 (50.0)	22 (50.0)	
Malocclusion			
Class I	21 (55.3)	17 (44.7)	>0.05
Class II	5 (35.7)	9 (64.3)	

*p-values calculated using Independent t-test and Chi-square test.

FRC= Fiber-reinforced composite.

MSW= Multistranded stainless steel wire.

Table 2 shows the comparison of plaque index (PI), calculus index (CI), gingival index (GI) and bleeding on probing (BOP) between the two groups of retainers. Mean (SD) values were assessed at baseline (T0), three months (T1), six months (T2), nine months (T3) and twelve months (T4) of insertion. At baseline (beginning of the trial), both groups exhibited similar healthy periodontal status. It can be seen that the health of periodontium deteriorated with the passage of time, from T1 to T4, in both groups of retainers. Moreover, Group 1 (FRC retainers) showed worse periodontal status than Group 2 (MSW retainers); however, there were no statistically significant differences between the two groups. 

**Table 2:** Comparison of plaque index (PI), calculus index (CI), gingival index (GI) and bleeding on probing (BOP) between two groups of retainers.

Mean (SD)					
Time interval	T0	T1	T2	T3	T4
PI					
Group 1	-	0.46 (0.65)	0.7 (0.62)	1.04 (0.66)	1.23 (0.65)
Group 2	-	0.23 (0.51)	0.6 (0.70)	0.9 (0.59)	1.12 (0.59)
p-value	-	> 0.05	> 0.05	> 0.05	> 0.05
CI					
Group 1	-	0.12 (0.43)	0.12 (0.43)	0.23 (0.51)	0.38 (0.57)
Group 2	-	0.00 (0.00)	0.15 (0.46)	0.23 (0.43)	0.38 (0.57)
p-value	-	> 0.05	> 0.05	> 0.05	> 0.05
GI					
Group 1	-	0.31 (0.55)	0.65 (0.69)	1.04 (0.66)	1.15 (0.68)
Group 2	-	0.15 (0.37)	0.5 (0.51)	0.81 (0.5)	0.92 (0.4)
p-value	-	> 0.05	> 0.05	> 0.05	> 0.05
BOP					
Group 1	-	0.08 (0.27)	0.23 (0.43)	0.42 (0.5)	0.5 (0.5)
Group 2	-	0.00 (0.00)	0.19 (0.4)	0.27 (0.45)	0.42 (0.5)
p-value	-	> 0.05	> 0.05	> 0.05	> 0.05

p-values calculated using Mann-Whitney test. Group 1 = FRC. Group 2 = MSW. T0 = baseline. T1 = 3 months after insertion. T2 = 6 months after insertion. T3 = 9 months after insertion. T4 = 12 months after insertion.

## DISCUSSION

In this randomized trial, the effects of two frequently used fixed lingual retainers on the periodontium health were determined and compared, using different indices. All cases selected were treated non-extraction in the mandibular arch, to minimize the biasness due to difference in treatment philosophy. When baseline demographic factors such as age, sex and type of malocclusion were compared, no statistically significant differences were found between the two groups, confirming that these factors did not affect the results of the study. 

FRC is accessible in varying widths and forms, including braided and woven polyethylene fibers. In this study, woven polyethylene fibers were used. 

The results of this study indicate that at the beginning of the trial (T0) both groups presented similar healthy periodontal status, suggesting adequate oral hygiene at the end of fixed orthodontic treatment. However, a continuous deterioration of periodontal health after placement of fixed lingual retainers was observed in the test period. Prolonged use of these retainers favor and attract plaque, lead to calculus deposition, gingival inflammation and bleeding on probing. Most probably, resin tags that extend gingivally favors the precipitation of biofilm, resulting in deposition of plaque and calculus.^18^ Therefore, excess resin and remnants should be removed from the surface and interproximal areas.

When the results were compared between the two groups of retainers, no significant differences were established between them. When plaque index was compared, plaque deposits were detected in both groups on probing the gingival margin (score 1) at T1 and T2, followed by visible plaque deposits on the surfaces (score 2) at T3 and T4. No copious amount of plaque (score 3) was detected in both groups. Calculus was seen only on two third of surfaces (score 1) from T1 to T4 in both groups of retainers. Separate specks or uninterrupted bands of subgingival calculus were not noticed. When gingival index was compared between groups, Group 1 (FRC) indicated mild inflammation with minor alteration in color and surface at T1 and T2, and moderate inflammation with reasonable glazing, redness, edema and hypertrophy at T3 and T4. Group 2 (MSW) exhibited mild inflammation, with minor alteration in color and surface throughout. Bleeding on probing was detected in both the groups. 

The present findings are in accordance with the study conducted by Artun et al,^24^ who compared spiral and plain wire fixed retainers and reported no differences in plaque and calculus accumulation. Moreover, Booth et al^17^ concluded that periodontal health is compatible with stainless steel fixed retainer. In a study conducted by Heier et al,^25^ spiral wire fixed retainer was compared with a removal retainer, and the authors reported increased calculus deposition in fixed retainer after six months of insertion; however, there were no significant difference in gingival index between the two types. Al-Moghrabi et al^15^ compared the periodontal effects of mandibular fixed retainer and removable retainer, and reported increase in gingival inflammation and plaque deposition with both types of retainers. Rody et al^11^ reported elevated levels of plaque and increased gingivitis in patients with fixed retainers, when compared to removable retainers. 

The present study was performed using a prospective design, allowing to evaluate the results in a reliable manner, as compared to retrospective studies, in which data is insufficient and unpredictable. Moreover, it was a multicentre study, involving the recruitment of subjects from different areas, thus providing a representative sample. 

The limitations included the follow-up time, which was limited to one year (It was not possible to extend the follow-up time of the tria). It was also not possible to blind the observers and the subjects, due to the visible nature of the intervention. Moreover, the findings of this study are limited to one year post-retention, therefore it could not be predicted whether the plaque and calculus deposits could lead to long-term periodontal problems. Other factors such as level of maintenance of oral hygiene and dietary habits could influence the results.

It is recommended to conduct similar studies with increased sample size and prolonged duration. Periodontal ligament width changes and bone level should also be evaluated in future. It is also suggested to evaluate the effects of other types of fiber-reinforced composites, such as nano-filled composites.

## CONCLUSION

The results of the study indicate that there is no significant difference on the health of periodontium between the FRC and MSW fixed retainers, therefore, the null hypothesis was accepted. Both types of mandibular retainers can be used alternatively. Mandibular fixed lingual retainers deteriorate the health of periodontium; therefore, routine checkup visits should be planned. 
